# Editorial: Exploring key pathways in the progression of gastrointestinal diseases based on metabolic reprogramming and developing drugs targeting metabolism

**DOI:** 10.3389/fimmu.2026.1808165

**Published:** 2026-02-26

**Authors:** Jilin Jia, Zimo Jia, Bing Yang

**Affiliations:** 1 Graduate School of Peking Union Medical College, National Research Institute for Family, Beijing, China; 2 Key Laboratory of Carcinogenesis and Translational Research (Ministry of Education), Department of Lymphoma, Peking University Cancer Hospital and Institute, Beijing, China; 3 Department of Cell Biology, College of Basic Medical Sciences, Tianjin Medical University, Tianjin, China; 4 Department of Medical and Nursing Science, International School, Krirk University, Bangkok, Thailand

**Keywords:** drug resistance, drug target, gastrointestinal disease, metabolic reprograming, tumor microenvironment

Gastrointestinal neoplasms continue to pose a serious threat to public health due to the high prevalence of highly lethal malignancies (1). These cancers can affect the esophagus, stomach, colon, liver, and pancreas. Patients often present asymptomatically, which leads to delays in diagnosis.

Significant advances have been made in cancer treatment over the past several decades, including the development of chemotherapy, targeted therapies, and immunotherapies.

However, resistance remains a significant challenge, with aberrant metabolism being recognized as one of the emerging hallmarks of cancer (2). As a heterogeneous disease, cancer retains its capacity to develop and progress through genetic and molecular changes (3).

The metabolism of these cells is ingeniously modified so that accelerated proliferation can be facilitated, apoptosis can be evaded, and even drug resistance can be developed. Recently, metabolic alterations have proven to be important in primary tumors, such as the Warburg effect (aerobic glycolysis), glutamine addiction, and reprogramming of lipid metabolism (4, 5).

Metabolic reprogramming of tumors may also interact with the tumor microenvironment (TME) by secreting metabolites such as lactate and succinate, thereby promoting immune suppression, angiogenesis, and stromal remodeling. These processes play an important role in the metastasis of gastrointestinal tumors, either directly or indirectly (6).

This present editorial introduces the comprehensive collection of articles that have hitherto been published in Frontiers in Immunology. The present volume is part of the Cancer Immunity and Immunotherapy Research Topic.

*ZIC2 drives colorectal cancer progression by regulating QPRT-mediated cell migration*, by Zheng et al. The present study investigates the expression and clinical significance of ZIC2 in cancer. This study is achieved through the molecular feature analysis of TCGA cohort data, GEO datasets (GSE39582 and GSE139555), and a CRC_10x spatial transcriptomics dataset.CRC_10x spatial transcriptomics dataset.

*GPR35-mediated metabolic reprogramming promotes tumorigenesis in digestive cancers* by Wang et al. This review examines the current understanding of the role of G protein-coupled receptor 35 in metabolic reprogramming, highlighting its regulatory functions in glucose, lipid, amino acid, and microbial metabolite metabolism.

*Targeting asparagine potentiates anti-PD-L1 immunotherapy in gastric cancer by enhancing CD8+ T cell anti-tumor response* by Ge et al. The author focuses on the key role of asparagine in the gastric cancer immune microenvironment and anti-PD-L1 therapy. They found that targeting asparagine could enhance the anti-tumor activity of anti-PD-L1 therapy, but this effect was significantly reduced by the depletion of CD8+ T cells.

*Metabolic syndrome in colorectal cancer liver metastasis: metabolic reprogramming and microenvironment crosstalk* by Ma et al. The review summarizes the recent progress in understanding the metabolic changes in colorectal cancer liver metastasis, particularly concerning metabolic reprogramming.

*Decoding the hypoxic tumor microenvironment in colorectal cancer for prognostic modeling and therapeutic target discovery* by Duan et al. The paper provides a new perspective on the impact of oxygen levels on the diversity of colorectal cancer. Additionally, the author discovered that GIPC2 has the potential to serve as both a biomarker and a therapeutic target.

*Clinical application prospects of traditional Chinese medicine as adjuvant therapy for metabolic reprogramming in colorectal cancer* by Liu et al. This review summarizes the traditional Chinese medicine approach to colorectal cancer. This could improve our understanding of the metabolic mechanisms involved in treating colorectal cancer.

*Lipid metabolic reprogramming in colorectal cancer: mechanisms and therapeutic strategies* by Liu et al. This review elucidates the contemporary comprehension of the fundamental and aberrant mechanisms of lipid metabolism in colorectal cancer.

*Sphingosine-1-phosphate stimulates colorectal cancer tumor microenvironment angiogenesis and induces macrophage polarization via macrophage migration inhibitory factor* by Wu et al. The present study focuses on the function of sphingosine-1-phosphate within the tumor microenvironment and angiogenesis.

*Endostatin-based anti-angiogenic therapy and immune modulation: mechanisms and synergistic potential in cancer treatment* by Sun et al. This review provides an overview of the extant literature regarding the mechanisms through which endostatin exerts its effects, including its capacity to inhibit angiogenesis and modulate the immune response. Further evaluations are made of the clinical efficacy of the treatment across solid tumors, with innovative strategies also being explored for the purpose of overcoming translational barriers.

*Mechanism of action and therapeutic value of anoctamin-1 in gastrointestinal cancers* by Zhang et al. The present review principally concentrates on the function of ANO1 in gastrointestinal cancers, with a view to supporting enhancements in therapeutic strategies for cancer diagnosis and treatment.
